# Solid-phase enrichment and analysis of electrophilic natural products

**DOI:** 10.3762/bjoc.13.43

**Published:** 2017-03-02

**Authors:** Frank Wesche, Yue He, Helge B Bode

**Affiliations:** 1 Merck Stiftungsprofessur für Molekulare Biotechnologie, Fachbereich Biowissenschaften, Goethe Universität Frankfurt, Max-von-Laue-Strasse 9, D-60438 Frankfurt am Main, Germany; 2 Buchmann Institute for Molecular Life Sciences (BMLS), Goethe Universität Frankfurt, Max-von-Laue-Strasse 15, D-60438 Frankfurt am Main, Germany

**Keywords:** azides, click chemistry, enrichment, electrophilic natural products, epoxides, glidobactin, *Photorhabdus*, stilbenes

## Abstract

In search for new natural products, which may lead to the development of new drugs for all kind of applications, novel methods are needed. Here we describe the identification of electrophilic natural products in crude extracts via their reactivity against azide as a nucleophile followed by their subsequent enrichment using a cleavable azide-reactive resin (CARR). Using this approach, natural products carrying epoxides and α,β-unsaturated enones as well as several unknown compounds were identified in crude extracts from entomopathogenic *Photorhabdus* bacteria.

## Introduction

Microorganisms are a major source for novel natural products and the subsequent development of new drugs for all kinds of applications [1–2]. For example, the discovery of the fungal natural product cyclosporine as an immunosuppressant drug facilitated modern organ transplantation [3–4].

The increasing sensitivity of analytical methods, especially in mass spectrometry, enables the detailed analysis of various natural product producing microbes. Much more compounds have been identified than originally thought, but often these are produced only at a very low level. This is also reflected by the genome sequences of bacteria and fungi that often encode numerous biosynthesis gene clusters (BGC) with most of the corresponding natural products unknown for several reasons: these BGCs are silent under standard laboratory conditions [5], the compounds are too labile for isolation or they are produced in amounts still below the detection limit of modern mass spectrometers.

Therefore it is desirable to have multiple and complementary methods available that allow the detection of several different natural product classes. Besides the traditional chemical screening and bioactivity-guided isolation, the exploitation of the inherent properties of natural products is also feasible. Consequently, simple functional groups of natural products like dehydroalanine [6–7], ketones, aldehydes [8–9], carboxylic acids [8–9], amines [8–10], thiols [8–9], alcohols [11], epoxides [12], terminal alkynes [13–14] and azides [15] can be targeted to introduce a label. Such labels might increase the visibility in UV or MS detection in liquid chromatography coupled to UV or mass spectrometry. Alternatively, natural products can be immobilized on reactive resins by making use of their chemical functionality and can be eluted after washing off all non-desired substances [8–9,11,13,15].

**Scheme 1:**
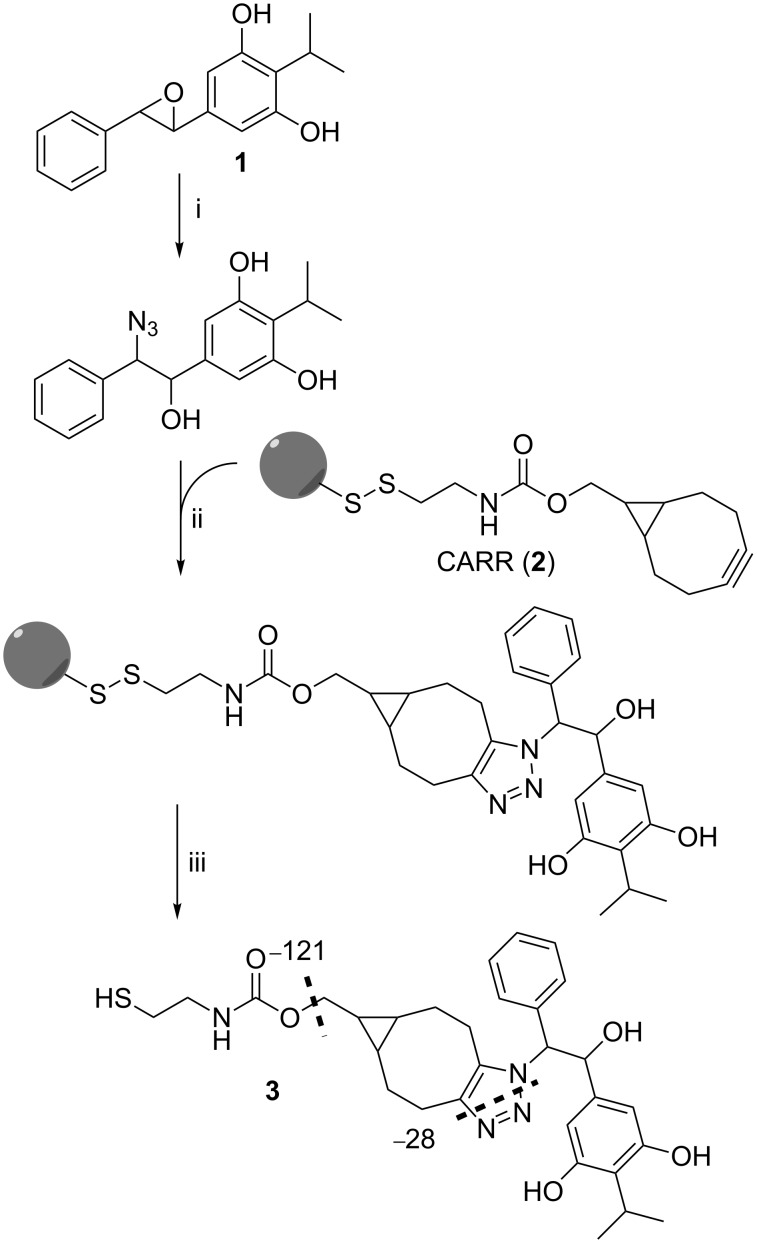
Principle of azidation of XAD extracts from *P. luminescens* TT01 containing **1** and subsequent azide enrichment with CARR (**2**). After the vicinal azido alcohol is covalently bound to the resin through an azide–alkyne cycloaddition, compound **3** is cleaved from the resin and analyzed by HPLC–MS. Reaction conditions: i) NaN_3_, NH_4_Cl, 80% MeOH in H_2_O, reflux overnight; ii) CARR (**2**), ACN, 55 °C, 1 h, then rt, overnight; iii) 5 mM TCEP in PBS/CHCl_3_/MeOH 1:5:10 (v/v/v), 1 h.

The recently introduced cleavable azide-reactive resin (CARR (**2**), Scheme 1) is such a resin able to react with a broad range of azides [15]. Thus, the metabolic fate of azide-containing biosynthesis intermediates or building blocks can be studied and natural products containing these azides can be identified.

Herein we describe the application of the CARR enrichment for the detection of electrophilic natural products using azide as the nucleophile. Indeed, we could detect epoxystilbene **1** and three glidobactins from different *Photorhabdus* strains and some of these compounds are produced in such low amounts that they are not detectable in standard crude extracts.

## Results and Discussion

**Figure 1:**
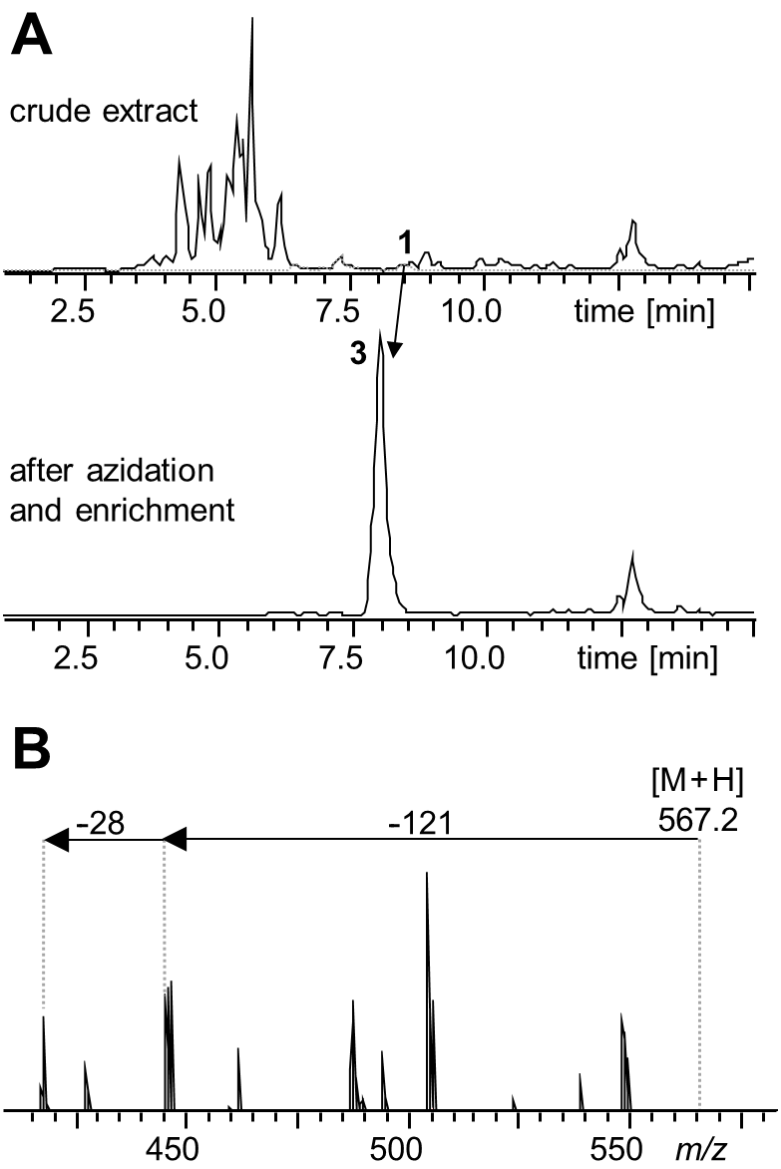
(A) HPLC–MS base peak chromatograms of a crude XAD extract of *P. luminescens* TT01 and after azidation and azide enrichment. The *t*_R_ of **1** is indicated in the crude extract. After enrichment, one distinct peak at 8.1 min corresponding to **3** is visible. (B) The characteristic MS^2^ fragmentation pattern of the derivatized azide **3** is shown highlighting the neutral loss of carbamate (−121) and dinitrogen (−28) as characteristic fragments for CARR adducts.

In order to evaluate whether the CARR approach is suitable for epoxide detection, we azidated commercially available *trans*-stilbene oxide as representative model compound for **1** affording the vicinal azido alcohol, 2-azido-1,2-diphenylethanol (Supporting Information File 1, Figure S2) [16–17]. The azidation was carried out with sodium azide and ammonium chloride in 80% MeOH under reflux overnight (Supporting Information File 1, Figure S2). The azido alcohol was then incubated with CARR (**2**) in acetonitrile overnight followed by extensive washing of the resin with methanol and dichloromethane. After disulfide-bond cleavage with a solution of tris(2-carboxyethyl)phosphine (TCEP) in a 1:5:10 mixture of phosphate-buffered saline (PBS)/chloroform/methanol at pH 7, the filtrate was analyzed by HPLC–MS. As expected the corresponding mass *m/z* 493.2 [M + H]^+^ of the cleaved cycloaddition product could be directly detected in the base-peak chromatogram (BPC) showing the characteristic fragmentation pattern of the CARR adducts (Supporting Information File 1, Figure S3) [15]. Additionally, the detection limit of the model epoxide was investigated in a complex environment. For this, defined amounts of *trans*-stilbene oxide were added to liquid Lysogeny Broth (LB) medium. The obtained methanolic Amberlite XAD-16 extracts were azidated, enriched and analyzed. Up to a final concentration of 5 µg/L (≈25 nmol/L) the epoxide could be detected (Supporting Information File 1, Figure S4). Following these results, we tested the method with an XAD extract of *P. luminescens* TT01 (Figure 1).

The obtained XAD extract was treated the same way as *trans*-stilbene oxide, and any potentially containing epoxide should be converted into the corresponding vicinal azido alcohol. Afterwards the azidated extract was incubated with **2**, the disulfide bond was cleaved and the filtrate subsequently analyzed by HPLC–MS. To our surprise, the BPC showed one distinct peak at 8.1 min with a characteristic MS^2^ fragmentation pattern of a derivatized azide and a mass of *m/z* 567.2 [M + H]^+^. This mass corresponds to the calculated mass of **3** (Figure 1) derived from the derivatization of epoxystilbene **1**, an oxidized isopropylstilbene derivative from this strain. Since only a single peak could be seen within the chromatogram, we assume that the conjugate addition took place only on the less hindered position in epoxide **1** without formation of the other possible regioisomer. For further structural confirmation, *P. luminescens* TT01 was cultivated in ^13^C-labeled medium prior to the azidation and enrichment procedure indeed confirming the incorporation of 17 carbon atoms in **3** (Supporting Information File 1, Figure S5). Furthermore, the molecular formula of the cleaved azide–alkyne cycloaddition product **3** was confirmed by HPLC–HRMS (calcd mass: *m/z* 567.2636 [M + H]^+^, found: *m/z* 567.2635 [M + H]^+^, Δppm = 0.1). Compound **1** could hardly be detected in extracts from standard growth media but was detected from infected insects and media mimicking the insect hemolymph [18–20]. Clearly the biosynthesis of this compound is strictly regulated and moreover it is a highly labile compound that is probably rapidly degraded [21]. Only a very weak signal of *m/z* 271.1 [M + H]^+^ could be detected at 8.5 min, which can be associated with **1**.

Encouraged by these results and especially the high sensitivity of the method, the azidation was performed with XAD extracts of three additional *Photorhabdus* strains (*Photorhabdus* PB45.5, *Photorhabdus* PB 68.1 and *Photorhabdus temperata* subsp. *thracensis* DSM 15199). Here, an even lower amount of **3** could be detected in *Photorhabdus* PB 68.1 (Supporting Information File 1, Figure S6), whereas in *Photorhabdus* PB45.5 and *Photorhabdus temperata* subsp. *thracensis* DSM 15199 nothing was visible at all, suggesting that the appropriate gene for the biosynthesis of **1** is either missing or silent in these species.

**Scheme 2:**
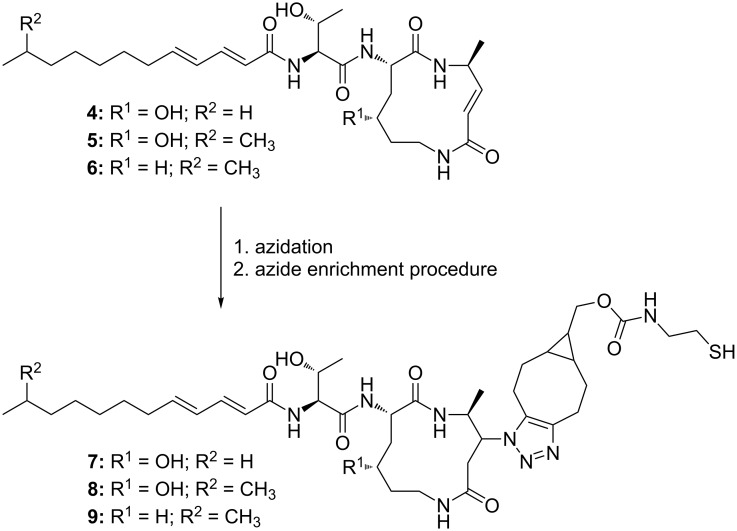
Structures of glidobactin derivatives (glidobactin A (**4**), cepafungin I (**5**) and luminmycin D (**6**)) before and after azidation and azide enrichment procedure (**7**, **8**, **9**). The MS^2^ spectrum indicates that azidation of glidobactins only took place on the reactive site that is also targeted by the proteasome (Supporting Information File 1, Figure S11) [24].

Upon a detailed look at the chromatograms, different masses with the characteristic fragmentation pattern of derivatized azides could be found (Table 1 and Supporting Information File 1, Figures S7–S9). The molecular formula obtained from HRMS data indicates that three glidobactin derivatives namely glidobactin A (**4**) [22], cepafungin I (**5**) [23–24] and luminmycin D (**6**) [25–26] were enriched (Scheme 2). Glidobactins are well-known proteasome inhibitors that react with a conserved threonine residue in the β5 subunit of the proteasome [24]. From *Photorhabdus* strains they have previously only been detected from infected insects [25], low-salt growth media [24] or by heterologous expression of the respective gene cluster in *E. coli* [26]. Their identification in this study was confirmed with pure glidobactin A (**4**) showing the same azide reactivity and retention time compared to **4** from the crude extract (Supporting Information File 1, Figure S10). The comparison of the MS/MS spectra of natural and azidated **4** revealed that the reaction took place at the ring-double bond that is also attacked by the threonine in the proteasome (Supporting Information File 1, Figure S11). To the best of our knowledge, this is the first detection of glidobactins in a supernatant of *Photorhabdus* under standard laboratory conditions, which points out once again the advantage of the enrichment step allowing the detection of otherwise barely detectable components in complex mixtures. A determination of the detection limit for pure glidobactin A (**4**) added into LB medium revealed a detection limit of 10 µg/L (≈20 nmol/L) (Supporting Information File 1, Figure S12) and was comparable to the detection limit of the epoxide (Supporting Information File 1, Figure S4).

**Table 1:** Additionally found masses in the tested strains, calculated molecular formulas of possible azide–alkyne cycloaddition products, and the molecular formulas of the putative parent compounds derived from subtraction of the azide and CARR-derived moiety (C_13_H_19_N_4_O_2_S).

Strain	Compd.	*t*_R_ min	[M + H]^+^ found	Calcd. molecular formula	[M + H]^+^ calcd.	Δppm	Molecular formula natural product	Natural product

*P. luminescens* TT01	**3**	8.1	567.2635	C_30_H_39_N_4_O_5_S	567.2636	0.1	C_17_H_18_O_3_	**1**

*Photorhabdus* PB 68.1	–	8.6	491.2103	C_27_H_31_N_4_O_3_S	491.2071	2.2	C_14_H_10_O	unknown
	–	8.0	493.2258	C_27_H_33_N_4_O_3_S	493.2268	2.0	C_14_H_12_O	unknown
	–	8.5	517.2483	C_26_H_37_N_4_O_5_S	517.2479	2.0	C_13_H_16_O_3_	unknown
	**3**	8.1	567.2635	C_30_H_39_N_4_O_5_S	567.2636	0.1	C_17_H_18_O_3_	**1**
	**7**	9.0	817.4630	C_40_H_65_N_8_O_8_S	817.4641	2.0	C_27_H_44_N_4_O_6_	**4**
	**8**	9.3	831.4801	C_41_H_67_N_8_O_8_S	831.4797	1.1	C_28_H_46_N_4_O_6_	**5**

*Photorhabdus* PB 45.5	–	10.7	551.3617	C_29_H_51_N_4_O_4_S	551.3626	1.5	C_16_H_30_O_2_	unknown
	**9**	9.8	815.4859	C_41_H_67_N_8_O_7_S	815.4848	1.3	C_28_H_46_N_4_O_5_	**6**
	–	10.1	819.5144	C_41_H_71_N_8_O_7_S	819.5161	2.0	C_28_H_50_N_4_O_5_	unknown

*Photorhabdus temperata* subsp*. thracensis* DSM 15199	–	8.0	493.2258	C_27_H_33_N_4_O_3_S	493.2268	2.0	C_14_H_14_O	unknown
	–	9.5	489.2525	C_25_H_37_N_4_O_4_S	489.2530	3.9	C_12_H_16_O_2_	unknown
	–	10.7	551.3617	C_29_H_51_N_4_O_4_S	551.3626	1.5	C_16_H_30_O_2_	unknown

## Conclusion

The combination of the reactivity-guided introduction of an azide functionality into electrophilic natural products and the subsequent azide enrichment on a solid phase facilitates the detection of epoxides and α,β-unsaturated enones in XAD extracts of *Photorhabdus.* Epoxystilbene (**1**) and glidobactins have never been observed before in XAD extracts of *Photorhabdus* grown under standard conditions. Most likely this is due to very low production levels of these compounds thus illustrating the power of this method. We deem that many more electrophilic compounds were just overlooked in the past due to their low concentrations. In combination with labeling experiments even the nature of the parent natural product could be revealed. Moreover, a possible scale-up of this procedure should enable the preparative purification of yet unidentified compounds as well as the structural confirmation of the identified structures. This approach can also be applied to extracts of other bacteria, fungi and plants and can give at least hints on new electrophilic natural products, where their reactivity against azide might also reflect their biological activity.

## Supporting Information

File 1 Materials, methods and supplementary figures.
