# Supplementary material for: Solid-phase enrichment and analysis of electrophilic natural products
Source: Beilstein J Org Chem. 2017 Mar 2;13:405–9. doi: 10.3762/bjoc.13.43 (PMC5355884; doi:10.3762/bjoc.13.43)
Supplement: File 1 — Materials, methods and supplementary figures. [file Beilstein_J_Org_Chem-13-405-s001.pdf]

Supporting Information
for
Solid-phase enrichment and analysis of electrophilic natural products

Frank Wesche[1], Yue He[1], and Helge B. Bode[*1,2]

Address: [1]Merck Stiftungsprofessur für Molekulare Biotechnologie, Fachbereich Biowissenschaften, Goethe Universität Frankfurt, Max-von-Laue-Strasse 9, D-60438 Frankfurt am Main, Germany and [2]Buchmann Institute for Molecular Life Sciences (BMLS), Goethe Universität Frankfurt, Max-von-Laue-Strasse 15, D-60438 Frankfurt am Main, Germany

Email: Helge B. Bode* - h.bode@bio.uni-frankfurt.de

*Corresponding author

Materials, methods and supplementary figures

Table of contents

General and synthetic procedures S2
Cultivation conditions S2
Preparation of XAD extracts S2
Azidation of XAD extracts S3
Azide enrichment procedure [3] S3
HPLC–MS analysis S3
Detection limit S4
Supplementary figures S4
References S12

General and synthetic procedures

Solvents and reagents were obtained from different suppliers and were used without furher purification. The ^1H NMR spectrum of the synthetic product was recorded on a Bruker AV250 spectrometer using $CDCl_3$ as solvent and internal standard (^1H NMR, $CHCl_3$: δ = 7.27 ppm).

2-Azido-1,2-diphenylethanol (2a) [1]

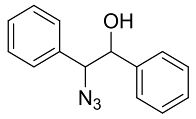

2a

To a solution of *trans*-stilbene oxide (32.3 mg, 0.17 μmol, 1 equiv) in 5 mL of 80:20 MeOH/water (v/v), sodium azide (32.1 mg, 0.5 μmol, 3 equiv) was added. Then, ammonium chloride (22.0 mg, 0.41 μmol, 2.5 equiv) was added and the solution was refluxed overnight. The mixture was diluted with iced water and extracted two times with dichloromethane (DCM). The combined organic layers were dried over $MgSO_4$ and purified with flash chromatography (Biotage SP1^{TM} flash purification system, Biotage, Uppsala, Sweden) using a SNAP cartridge KP-Sil 10 g and a 0–50% ethyl acetate in *n*-hexane gradient to afford 2-azido-1,2-diphenylethanol (11 mg, 28%) as a pale yellow oil. ^1H NMR (250 MHz, $CDCl_3$): δ 7.36-7.21 (m, 10 H), 4.84 (d, J = 6.7 Hz, 1 H), 4.69 (d, J = 6.7 Hz, 1 H), 1.99 (bs, 1 H) [2].

Cultivation conditions

All strains were inoculated into liquid Lysogeny broth (LB, pH 7.0) medium and grown at 30 °C with shaking at 200 rpm overnight. A 200 μL aliquot of overnight culture was transferred into 20 mL fresh LB medium containing 2% (v/v) of Amberlite XAD-16 resin (Sigma–Aldrich). Finally, the cultures were grown at 30 °C for 72 h at 200 rpm.

Preparation of XAD extracts

After 72 h of growth, bacteria and XAD were harvested by centrifugation and resuspended in 30 mL of MeOH. The extraction was carried out by continuous inversion XAD and

methanol for 1 h. After extraction, the methanol extracts were separated from XAD beads via filter paper and evaporated to dryness. Before analysis, the residue was redissolved in 1 mL of methanol. For labeling experiments, the *P. luminescens* TT01 strain was cultivated in 5 mL ^{13}C medium and handled the same way.

Azidation of XAD extracts

To the obtained XAD extract was added sodium azide (32.1 mg, 0.5 µmol, 3 equiv) and ammonium chloride (22.0 mg, 0.41 µmol, 2.5 equiv) in 5 mL of 80:20 MeOH/water (v/v) and the solution was refluxed overnight. The mixture was diluted with iced water and extracted with dichloromethane. The combined organic layers were dried over $MgSO_4$ and the solvent was evaporated.

Azide-enrichment procedure [3]

DMF-wetted CARR (**2**, 3 mg) was placed inside a syringe for solid-phase peptide synthesis and thoroughly washed with MeOH and DCM. The respective azidated product or azidated XAD extract was dissolved or redissolved in 1 mL ACN and then added. The suspension was heated to 55 °C for 1 h and then stirred overnight. Excess extract was then removed, and the resin was extensively washed with MeOH and DCM and subsequently incubated with 1 mL of a phosphate-buffered (pH 7) solution of tris(2-carboxyethyl)phosphine (TCEP, 5 mM) in PBS/$CHCl_3$/MeOH 1:5:10 (v/v/v) for 1 h. The resulting solution was collected and concentrated to dryness. The cleavage products were redissolved in ACN (200 µL) and analyzed directly by HPLC–MS.

HPLC–MS analysis

Five µL of the sample (crude extract, cleavage products) were injected and analyzed via HPLC–ESIMS by a Dionex UltiMate 3000 system coupled to a Bruker AmaZon X mass spectrometer with a ACQUITY UPLC™ BEH C18 column (130 Å, 2.1 mm × 100 mm, 1.7 µm particle size, Waters GmbH) at a flow rate of 0.4 mL/min for 16 min, using ACN and water supplemented with 0.1% formic acid (v/v) in a gradient ranging from 5 to 95% ACN. For detection, the positive mode with a scanning range from 100–1200 *m*/*z* was used. HRMS–ESI analysis was carried out by using an internal calibrant (10 mM sodium formate solution) and the same linear gradient from 5–95% ACN (0.1% formic acid). HR-masses were detected by a Bruker Impact II QTOF.

Detection limit

For determination of the detection limit, defined amounts of *trans*-stilbene oxide or **4** were added to 10 mL LB medium with 2% (v/v) of XAD resin resulting in final concentrations of 500, 100, 10, 2 and 1 µg/L. The flasks were shaken at 30 °C for 24 h at 200 rpm. Afterwards XAD was collected by centrifugation and resuspended in 10 mL MeOH. After extraction, the methanol extracts were separated from XAD beads via filter paper and evaporated to dryness. The resulting residue was azidated and enriched as described above. Figure S4 and Figure S12 show the EIC of expected masses of lowest detectable concentration of click-products compared to blanks (no compound added), and calculated signal-to-noise ratios (S/N). S/N was calculated according to equation (1).

$$S/N = \frac{2H}{h} \qquad (1)$$

Supplementary figures

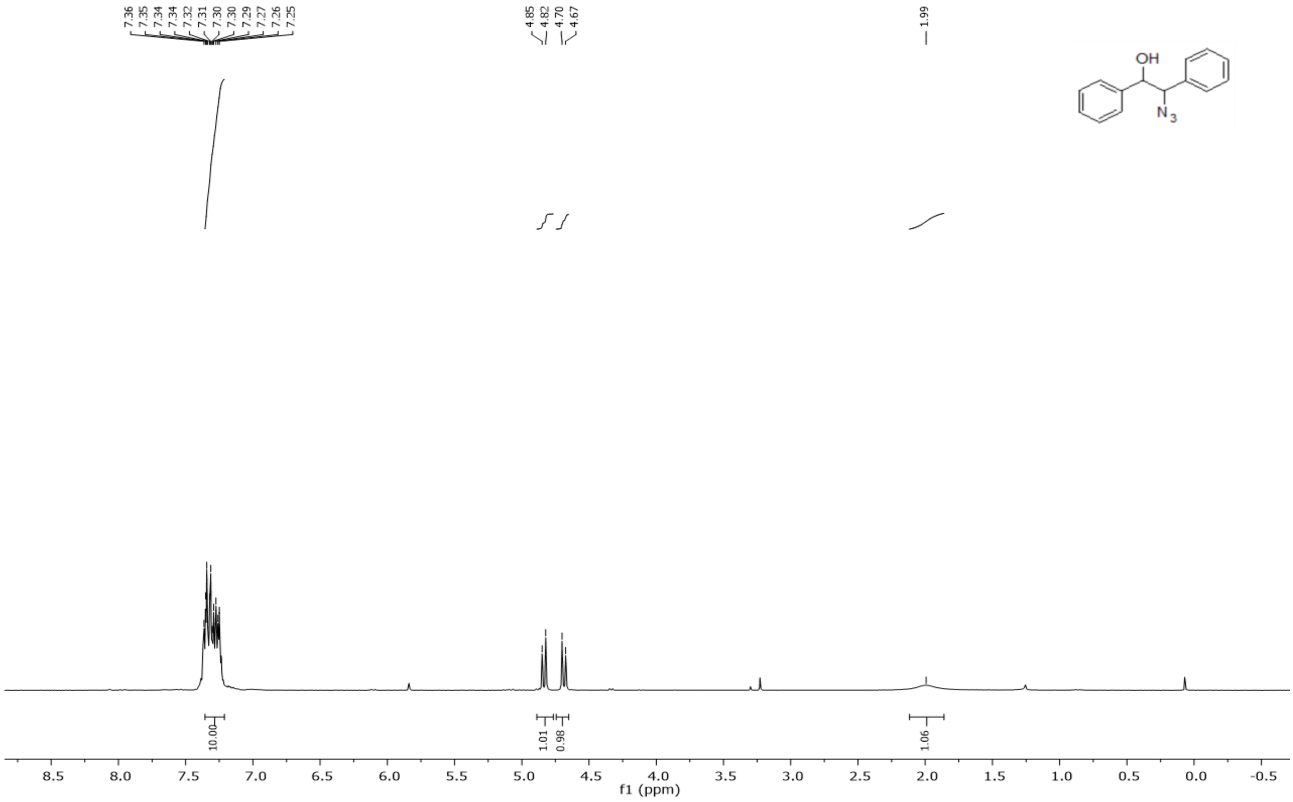

Figure S1: ^{1}H NMR (250 MHz, $CDCl_3$) spectrum of 2-azido-1,2-diphenylethanol.

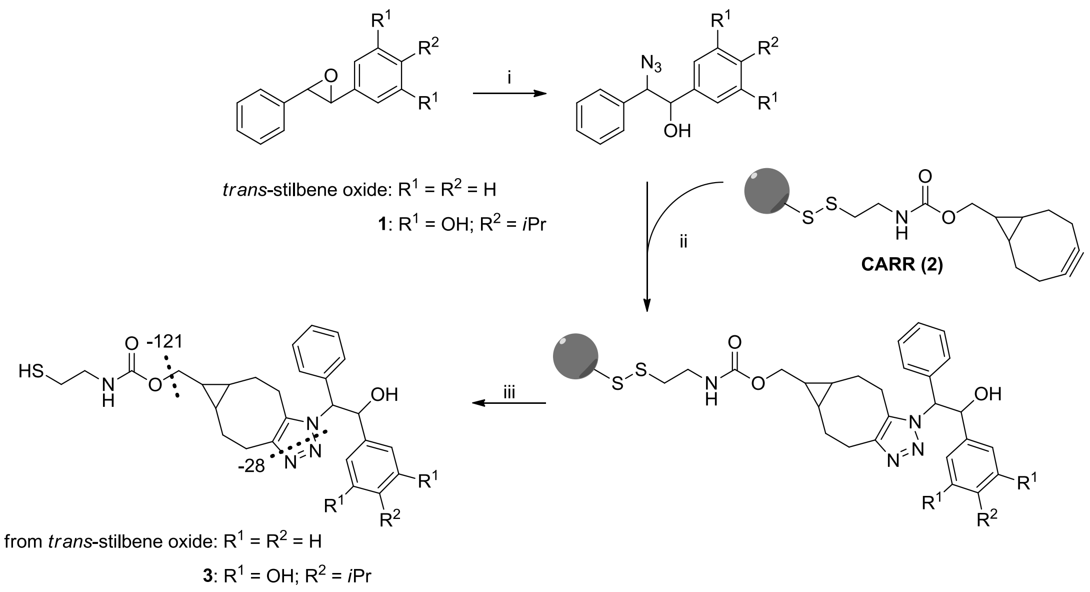

Figure S2: Azidation of *trans*-stilbene oxide and **1** to the corresponding vicinal azido alcohols and subsequent azide enrichment with CARR (**2**). Reaction conditions: i) NaN_3, NH_4Cl, 80% MeOH in H_2O, reflux overnight; ii) CARR (**2**), ACN, 55 °C, 1 h, then rt, overnight; iii) 5 mM TCEP in PBS/$CHCl_3$/MeOH 1:5:10 (v/v/v), 1 h.

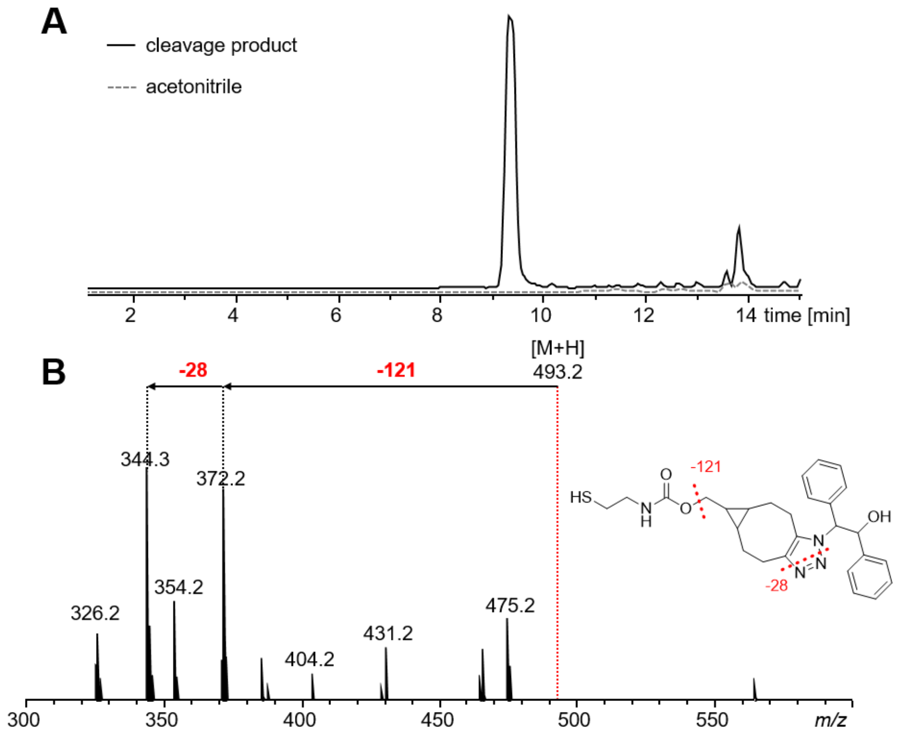

Figure S3: HPLC–MS base peak chromatogram (BPC) of cleavage product (A) and MS^2 spectrum at 9.3 min, the common losses of carbamate (−121) and dinitrogen (−28) are delineated (B).

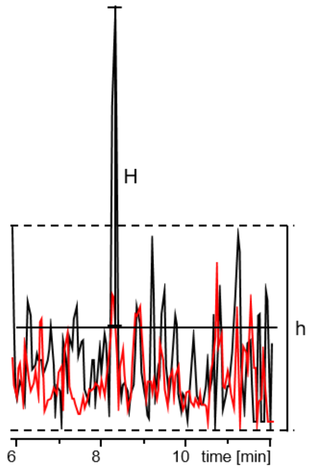

Figure S4: Determination of the detection limit for *trans*-stilbene oxide by HPLC–MS. EIC of cleavage product (*m/z* 493.2 $[M + H]^+$) after azidation and enrichment of XAD extract containing 5 µg/L (~25 nmol/L) of *trans*-stilbene oxide (black) compared to blank (red). S/N = 3.1 is shown.

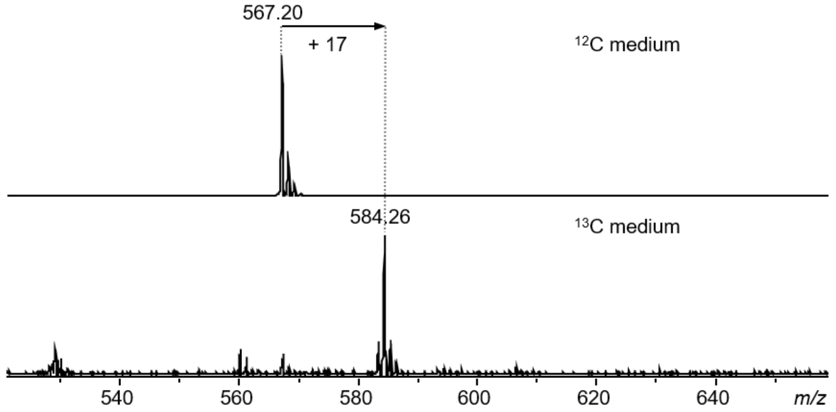

Figure S5: Incorporation of ^{13}C atoms in **3** after cultivation in ^{13}C medium, azidation and azide enrichment, proving that the expected 17 carbon atoms are incorporated.

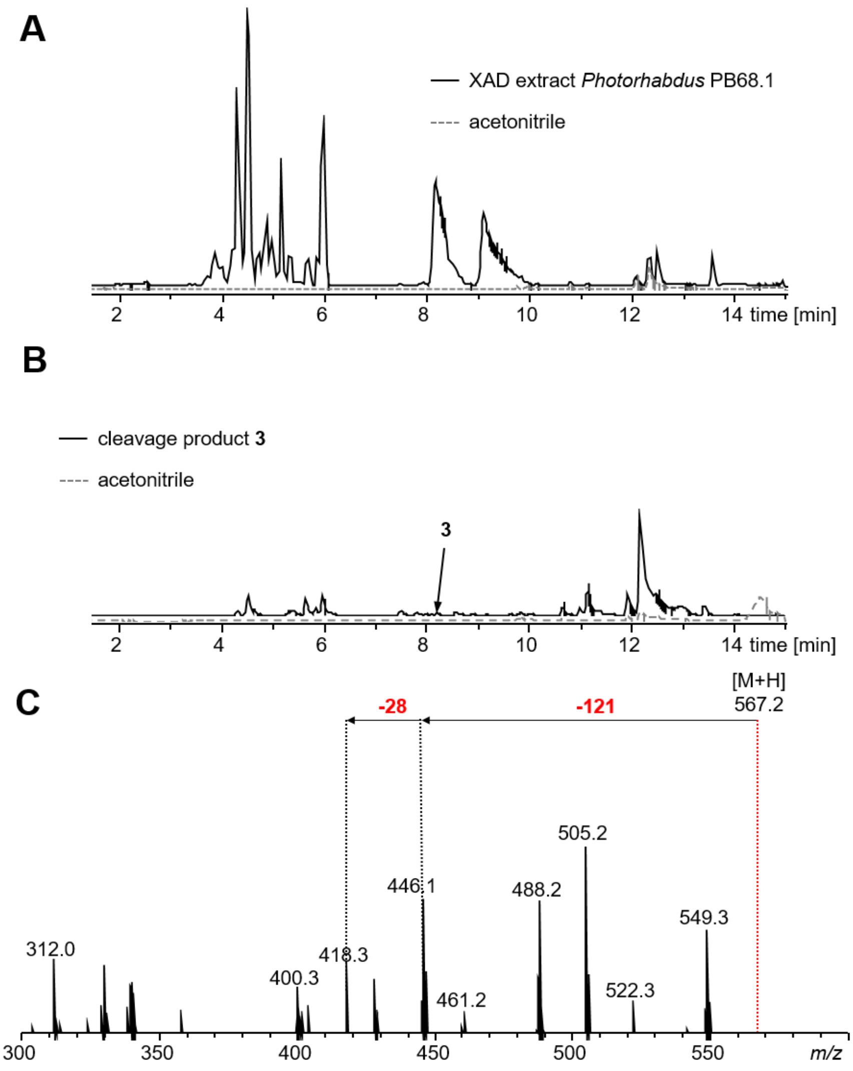

Figure S6: HPLC–MS, BPC of crude XAD extract of *Photorhabdus* PB68.1 (A); BPC of cleavage product **3** at 8.1 min after azidation of the crude extract and subsequent azide enrichment (B); MS^2 spectrum of **3**, the common losses of carbamate (−121) and dinitrogen (−28) are delineated (C).

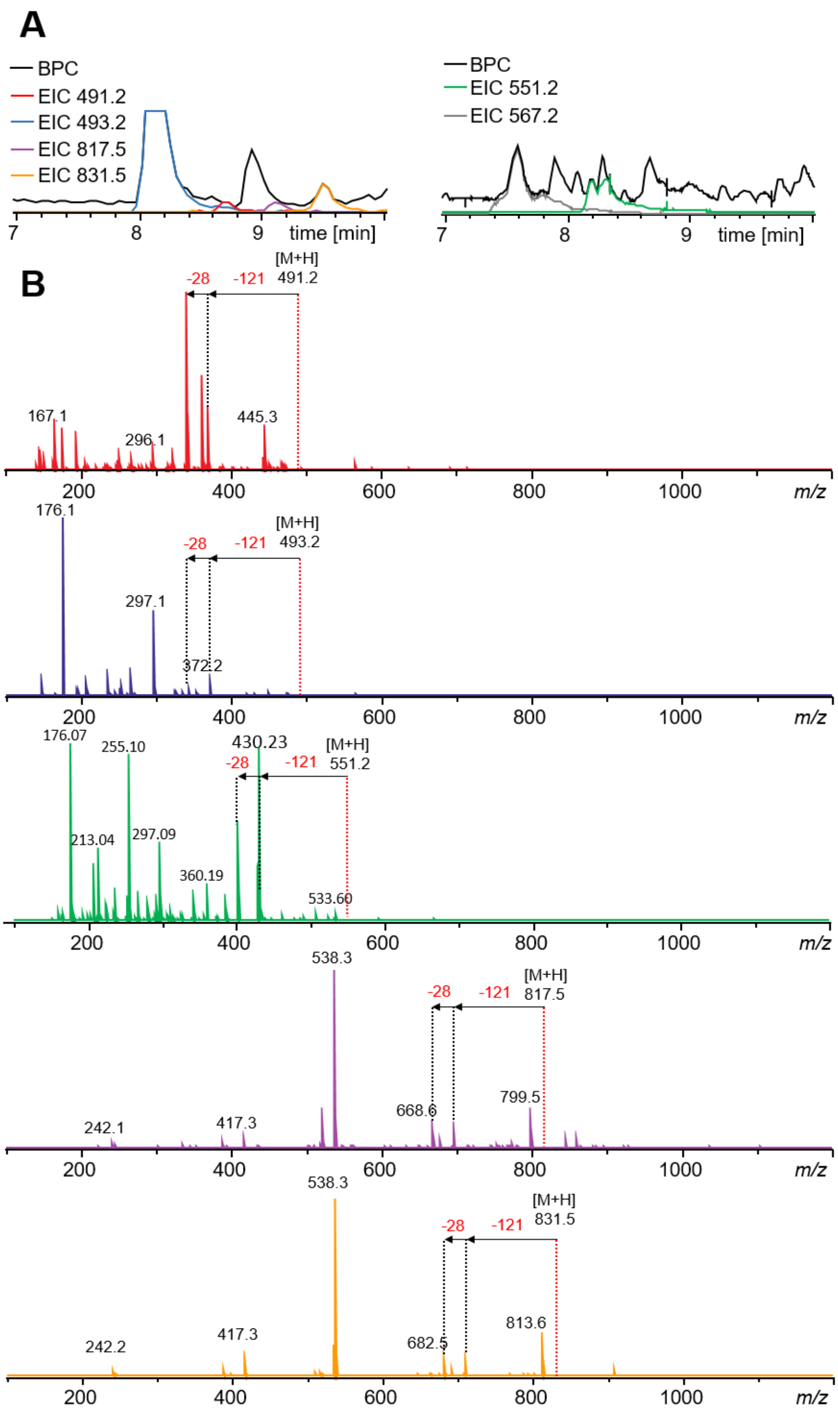

Figure S7: BPC and superimposed extracted ion chromatograms (EIC) of azidated and enriched XAD extract of *Photorhabdus* PB68.1 from two different experiments (A). MS^2 fragmentation of different compounds with the common losses of carbamate (−121) and dinitrogen (−28) are delineated (B). MS^2 of *m/z* 567.2 $[M + H]^+$ is already shown in Figure S6. From HPLC–HRMS of *m/z* 817.5 $[M + H]^+$, comparison of isolated, azidated and enriched glidobactin A (**4**) [4] could be assigned. The mass *m/z* 831.5 $[M + H]^+$ could be assigned to cepafungin I (**5**) [5] from the HR data. Due to their very low concentration or their poor ionizability, no compound can be seen prior to azidation and enrichment.

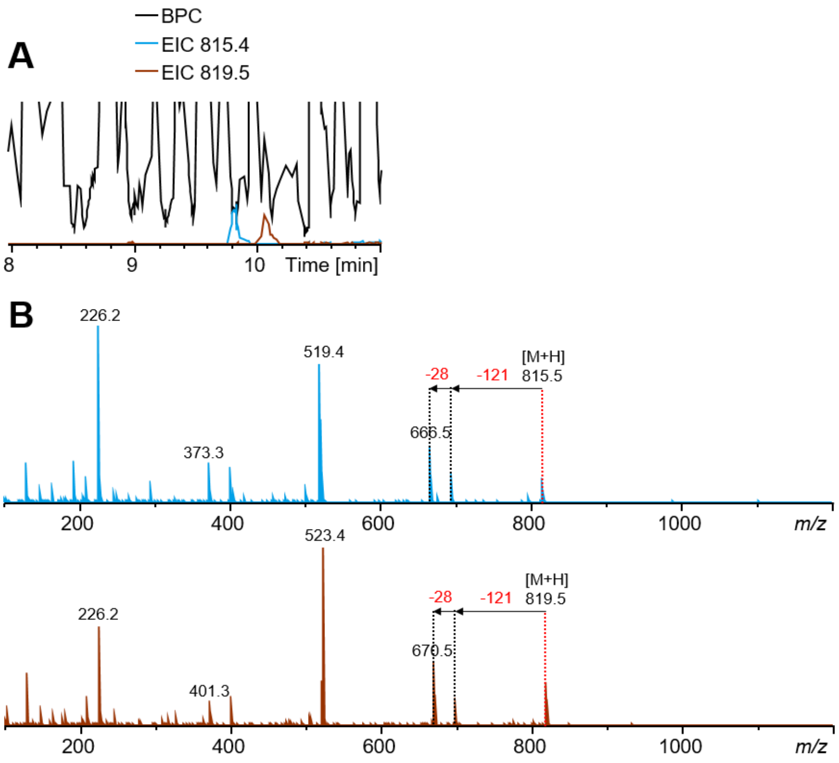

Figure S8: BPC and superimposed EICs of azidated and enriched XAD extract of *Photorhabdus* PB45.5 (A). MS^2 fragmentation of different compounds with the common losses of carbamate (−121) and dinitrogen (−28) are delineated (B). From HPLC–HRMS of *m/z* 815.5 $[M + H]^+$ it could be identified as luminmycin D (**6**) [6]. Due to their very low concentration or their poor ionizability, no compound can be seen before azidation and enrichment.

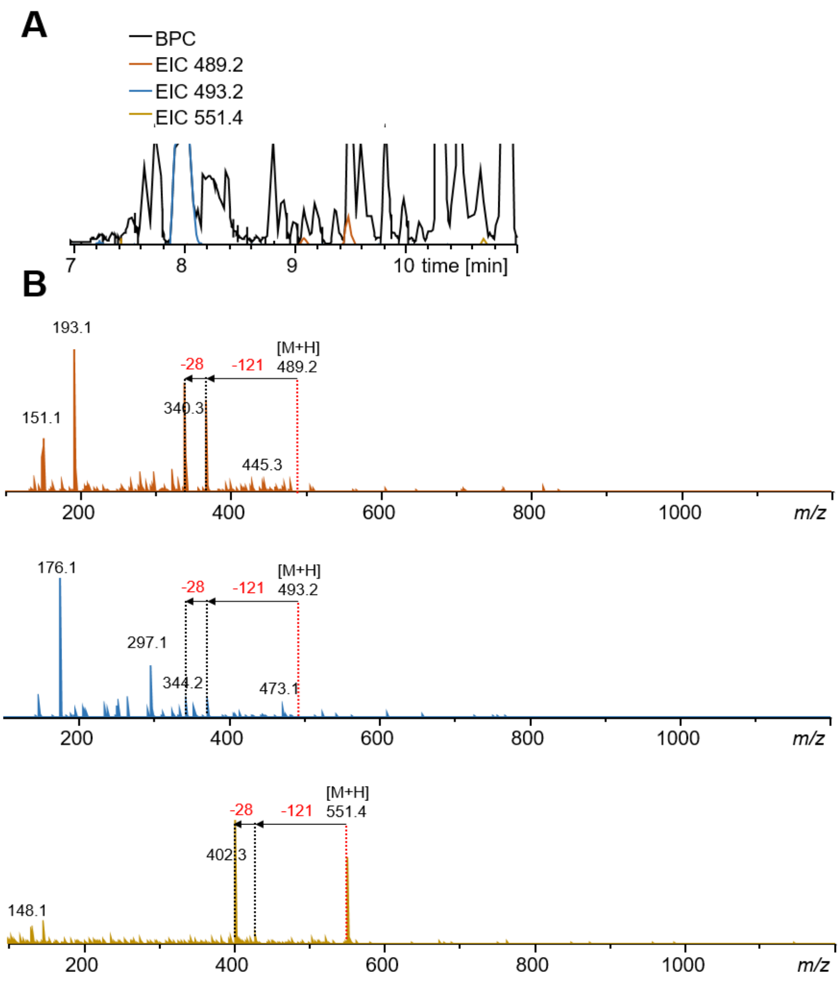

Figure S9: BPC and superimposed EICs of azidated and enriched XAD extract of *Photorhabdus temperata* subsp. *thracensis* DSM 15199 (A). MS^2 fragmentation of different compounds with the common losses of carbamate (−121) and dinitrogen (−28) are delineated (B). Due to their very low concentration or their poor ionizability, no compound can be seen before azidation and enrichment.

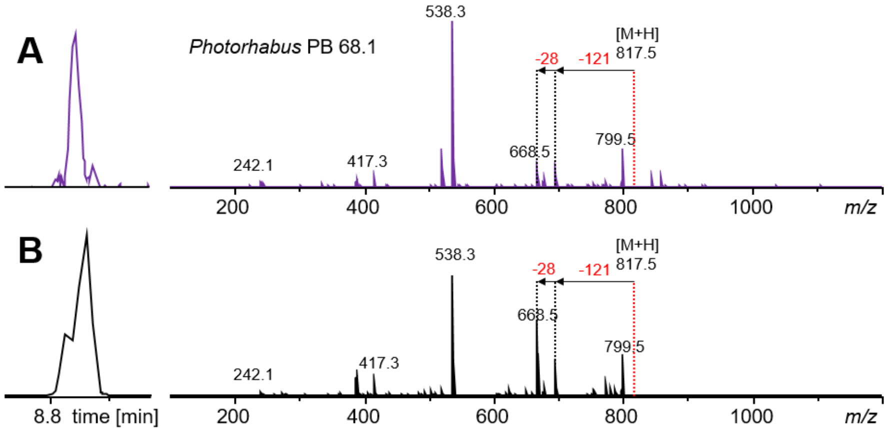

Figure S10: EIC and MS^2 fragmentation of azidated and enriched **4** in XAD extracts of *Photorhabdus* PB68.1 (A) and from pure compound **4** (B).

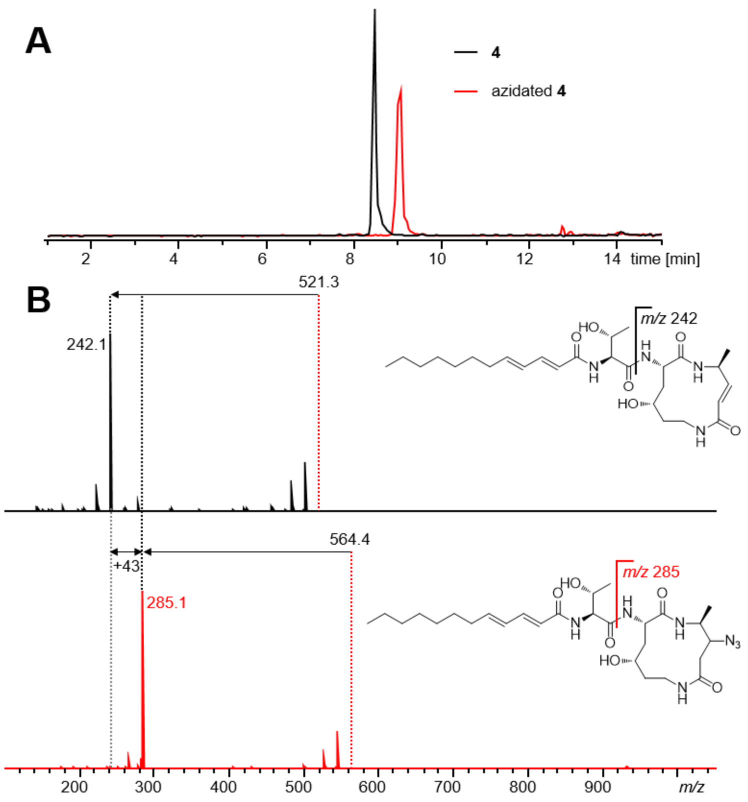

Figure S11: HPLC–MS, EIC of **4** (*m/z* 521.3 $[M + H]^+$) and azidated **4** (*m/z* 564.4 $[M + H]^+$) after azidation under given conditions. Masses of double conjugate addition products could not be observed (A). MS^2 spectra of **4** and azidated **4** illustrate that the conjugate addition of the azide (+43) only took place at the reactive site of the ring that is also targeted by the proteasome (B) [7].

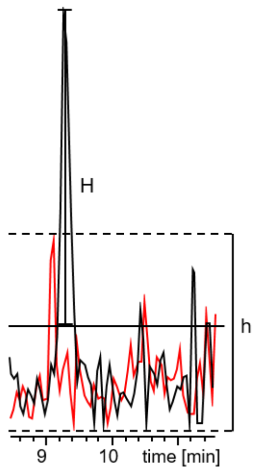

Figure S12: Determination of the detection limit of **4** from HPLC–MS, EIC of cleavage product **7** (*m/z* 817.4 $[M + H]^+$) after azidation and enrichment of XAD extract containing 10 µg/L (~20 nmol/L) **4** (black) compared to blank (red). S/N = 3.2.

References

1. Rowland, E. B.; Rowland, G. B.; Rivera-Otero, E.; Antilla, J. C. *J. Am. Chem. Soc.* **2007,** *129,* 12084–12085. doi:10.1021/ja0751779
2. Lupattelli, P.; Bonini, C.; Caruso, L.; Gambacorta, A. *J. Org. Chem.* **2003,** *68,* 3360–3362. doi:10.1021/jo034133p
3. Perez, A. J.; Wesche, F.; Adihou, H.; Bode, H. B. *Chem. Eur. J.* **2016,** *22,* 639–645. doi:10.1002/chem.201503781
4. Oka, M.; Nishiyama, Y.; Ohta, S.; Kamei, H.; Konishi, M.; Miyaki, T.; Oki, T.; Kawaguchi, H. *J. Antibiot.* (Tokyo) **1988,** *41,* 1331–1350. doi.org/10.7164/antibiotics.41.1331
5. Terui, Y.; Nishikawa, J.; Hinoo, H.; Kato, T.; Shoji, J. *J. Antibiot.* **1990,** *43,* 788–795. doi.org/10.7164/antibiotics.43.788
6. Theodore, C. M.; King, J. B.; You, J.; Cichewicz, R. H. *J. Nat. Prod.* **2012,** *75,* 2007–2011. doi:10.1021/np300623x
7. Stein, M. L.; Beck, P.; Kaiser, M.; Dudler, R.; Becker, C. F. W.; Groll, M. *Proc. Natl. Acad. Sci. USA* **2012,** *109,* 18367–18371. doi:10.1073/pnas.1211423109
